# PILOTING AN INTERGENERATIONAL ECOSYSTEM: THE GEN2GEN INNOVATION FELLOWSHIP AND RESULTS FROM YEAR 1

**DOI:** 10.1093/geroni/igac059.044

**Published:** 2022-12-20

**Authors:** Cal Halvorsen, Eunice Lin Nichols, Janet Oh

**Affiliations:** Boston College, Allston, Massachusetts, United States; Encore.org, San Francisco, California, United States; Encore.org, San Francisco, California, United States

## Abstract

Intergenerational service has a long history in the U.S., highlighting the positive health and well-being outcomes of bringing the generations together. In recent years, private foundations have increased their focus on intergenerational initiatives, yet the creation of an “intergenerational ecosystem” that is designed to spark, grow, and support new intergenerational initiatives has not been nationally tested. In response, Encore.org launched the Gen2Gen Innovation Fellowship in 2020 to pilot and hone a 9-month fellowship model that supports a diverse group of leaders of intergenerational initiatives. To assess the impact of the Fellowship, we employed a mixed-methods approach with subjective and objective data sources, including interviews and monthly and post-Fellowship surveys. Among the first cohort of 15 Fellows, results suggest that the cohort-style model encouraged peer-to-peer support and that the Fellowship positively influenced their intergenerational efforts. We conclude by describing lessons learned to inspire future efforts to strengthen the intergenerational ecosystem.

